# Maternal-Fetal HLA Compatibility in Uncomplicated and Preeclamptic Naturally Conceived Pregnancies

**DOI:** 10.3389/fimmu.2021.673131

**Published:** 2021-05-13

**Authors:** Liseanne J. van ‘t Hof, Naomi Schotvanger, Geert W. Haasnoot, Carin van der Keur, Dave L. Roelen, Lisa E. E. L. O. Lashley, Frans H. J. Claas, Michael Eikmans, Marie-Louise P. van der Hoorn

**Affiliations:** ^1^ Department of Obstetrics and Gynaecology, Leiden University Medical Center, Leiden, Netherlands; ^2^ Department of Immunology, Leiden University Medical Center, Leiden, Netherlands

**Keywords:** human leukocyte antigen, HLA compatibility, pregnancy, preeclampsia, killer-cell Immunoglobin-like receptor, reproductive immunology

## Abstract

**Introduction:**

In pregnancy, the mother and fetus differ in HLA antigens, and yet the maternal immune system generally tolerates the fetus. KIR receptors expressed by maternal uterine NK cells at the maternal-fetal interface directly interact with HLA-C on extravillous trophoblast cells for optimal placental development. In this study, we aimed to determine whether there is a preferential selection for HLA compatibility and specific KIR/HLA-C combinations in uncomplicated and preeclamptic naturally conceived pregnancies compared to what would be expected by chance.

**Methods:**

Genotyping for maternal and fetal HLA-A, -B, -C, -DR, and -DQ, and maternal KIR was performed for 451 uncomplicated pregnancies and 77 pregnancies complicated with preeclampsia. The number of HLA antigen (mis)matches between mother and fetus was calculated and compared to expected values obtained by randomization of the HLA haplotype, inherited from the father, over the existing maternal haplotype of the fetuses. A similar methodology was executed for analysis of the KIR/HLA-C data (n=309).

**Results:**

In uncomplicated pregnancies, the degree of maternal-fetal HLA matching was not different than expected-by-chance values. In preeclamptic pregnancies, the degree of maternal-fetal HLA matching was different in observed compared to expected-by-chance values (p=0.012). More specifically, the degree of maternal-fetal matching of HLA-C was higher in the actual preeclamptic pregnancies than was expected-by-chance (p=0.007). Preeclamptic pregnancies showed an overall tendency towards higher maternal-fetal HLA compatibility, for total HLA matches (p=0.021), HLA class I (p=0.038) and HLA-C (p=0.025) compared to uncomplicated pregnancies.

**Conclusion:**

The data suggest that there is no preferential selection of maternal-fetal HLA compatibility in uncomplicated pregnancies. In contrast, increased total HLA, HLA class I and, especially, HLA-C compatibility is associated with preeclampsia, suggestive for a role of HLA mismatches in immune regulation leading to uncomplicated pregnancy.

## Introduction

In pregnancy, a successful development of the semi-allogeneic placenta and fetus within the womb requires modulation of the maternal immune system. Encounter of cells and immunogenic molecules from the fetus mainly occurs at the fetal-maternal interface (1–3). The trophoblast expresses a specific HLA profile that constitutes classical polymorphic HLA-C as well as non-classical oligomorphic HLA-E, -F, and -G, while lacking the other HLA molecules (4). The expressed HLA molecules play a crucial role in placentation and pregnancy outcome, as these mediate contact between the extravillous trophoblast (EVT) and maternal decidual leukocytes (1).

Especially the interaction of maternal uterine NK (uNK) cells with the invading EVT is essential for implantation, placentation and spiral artery remodeling (5, 6). The killer-cell immunoglobin-like receptor (KIR) on uNK cells can directly interact with HLA-C molecules expressed on the surface of EVTs to enhance these processes, mainly through the release of cytokines. The interaction of maternal KIR with fetal HLA-C is crucial for the success of the pregnancy, as specific combinations are associated with enhancement or disruption of placentation. Specifically, Hiby et al. found that the KIR B haplotype has a protective effect in reproduction (7). Data from the same research group indicates that the combination of the KIR AA genotype with the paternally inherited fetal HLA-C2 genotype causes inhibition of uNK cells and subsequent disturbance of placentation (8). In addition, the lack of uNK activation is associated with the risk of severe pregnancy complications such as preeclampsia (8).

Although studied to a much lesser extent, it has been shown that decidual T cells can also express NK-associated KIR receptors, suggesting a similar interaction of such T cells with invading EVTs (9). While direct CD4^+^ T cell recognition seems impossible due to lack of HLA class II expression by trophoblasts, maternal-fetal HLA-C mismatching during pregnancy is associated with increased numbers of CD4^+^CD25^dim^ activated T cells and functional CD4^+^CD25^bright^ regulatory T cells at the maternal-fetal interface (10). These findings are suggestive for a role of indirect allorecognition in the local immune regulation.

Even though the trophoblast has a limited profile of HLA expression, the degree of complete HLA and HLA subclasses I and II compatibility between mother and child has been associated with pregnancy outcome and risk of complications. A high level of HLA compatibility has been linked to preeclampsia and to recurrent pregnancy losses, although both relationships seem to be based on biased studies or inconsistent results (11, 12).

In oocyte donation pregnancies, the significance of a certain degree of maternal-fetal HLA compatibility for immune modulation and pregnancy outcome has been suggested. Oocyte donation pregnancies are characterized by an increased level of HLA mismatching and consequently a greater immunogenic dissimilarity compared to naturally conceived pregnancies. Previous research showed that there is a selective advantage of HLA compatibility, to a similar degree as the one observed in naturally conceived pregnancies, in successful oocyte donation pregnancies (13). In addition, we found a strong relationship between the number of HLA class II mismatches and the occurrence of preeclampsia in oocyte donation pregnancies (14). Furthermore, we recently demonstrated that the total amount of HLA mismatches and that of HLA class I mismatches is associated with expression of immune modulatory molecules CD200 in uncomplicated oocyte donation pregnancies, suggestive of a higher compensatory need (15). Thus, a certain degree of maternal-fetal HLA compatibility seems to translate in a successful outcome of pregnancy in oocyte donation pregnancies.

As the implications of the extent of maternal-fetal HLA compatibility are not yet established in naturally conceived pregnancies, we aimed to determine whether there is a preferential selection for HLA compatibility and specific KIR/HLA-C combinations in naturally conceived pregnancies compared to what would be expected by chance. Furthermore, we compared observed and expected HLA compatibility in a cohort of women with a pregnancy complicated with preeclampsia.

## Material and Methods

### Subjects

451 uncomplicated naturally conceived pregnancies and 77 naturally conceived pregnancies complicated with preeclampsia were included in this retrospective observational study. Within 24h of delivery, maternal peripheral blood samples and fetal umbilical cord blood samples were collected. All samples were collected at the Leiden University Medical Center (LUMC) from June 1993 up until October 2019.

Exclusion criteria consisted of pregnancies conceived after artificial technologies, multiplet pregnancies, pre-existing high diastolic blood pressure, maternal autoimmune diseases, pregnancies with fetal chromosomal abnormalities, receipt of blood transfusions, organ transplantation in the medical history, and infection during pregnancy.

Clinical information on maternal age, gestational age, fetal gender, birth weight, gravidity, parity and highest gestational diastolic blood pressure was collected from the medical records. Subjects were included as uncomplicated cases if the pregnancy had no preeclampsia, HELLP, preterm birth, decreased birth weight, fetal growth restriction or infection.

Preeclampsia was defined according to the definition by the International Society for the study of Hypertension in Pregnancy (ISSHP) (16, 17). All selected cases (also those selected before the ISSHP definition of 2001) had co-existing proteinuria (≥0.3 g/l/24 h) and gestational hypertension (systolic blood pressure ≥140 mmHg and/or diastolic blood pressure ≥90 mmHg detected after 20 weeks of gestation) or worsening of pre-existing hypertension.

Informed consent was obtained from every mother. The study protocol was approved by the ethics committee of the LUMC with protocol number P16.048.

### HLA and KIR Typing

HLA genotyping was performed using the collected maternal peripheral blood and umbilical cord blood of all included cases. The Reverse Sequence Specific Oligonucleotides PCR technique was used to type the extracted DNA samples for HLA -A, -B, -C, -DR, and -DQ loci. The number of fetal-maternal HLA (mis)matches was calculated at the Dutch National Reference Laboratory for Histocompatibility Testing (LUMC). Compatibility for HLA class I and class II was determined at the second field level. The total number of antigen matches in these observed naturally conceived pregnancies ranges from 5 to 10. HLA loci showing homozygosity in both mother and child were counted as respectively 1 match and 0 mismatch.

For the KIR/HLA-C analysis, the HLA-C alleles were arranged into C1 and C2 groups. The C1 group consists of the following alleles: C*01, C*03, C*07, C*08, C*12, C*14, and C*1601. The C2 group consists of C*02, C*04, C*05, C*06, C*15, C*1602, C*17, and C*18.

Genotyping of maternal KIR was performed for 309 women using the real-time PCR technique with sequence specific primers. DNA was amplified and labeled with SYBRgreen (BioRad). Eleven KIR genes were typed: 2DL1, 2DL2, 2DL3, 2DL5, 2DS1, 2DS2, 2DS3, 2DS4, 2DS5, 3DL1, and 3DS1. The KIR B haplotype was assigned based on the presence of one or more of the following genes: 2DL2, 2DL5, 2DS1, 2DS2, 2DS3, 2DS5, 3DS1. The KIR AA haplotype was assigned when these specific genes were absent.

### Statistical Analysis

Maternal and fetal HLA genetic frequencies were examined for Hardy-Weinberg equilibrium (Hardy, 1908; Weinberg, 1963) with Pypop Software 0.7.0, to test whether the cohort was a reliable representation of the general population. For the analysis through the Hardy-Weinberg equilibrium, the following principle was applied: the allele frequencies in a population will remain constant under specific circumstances; in the presence of random mating and in the absence of migration and mutation.

The expected number of HLA (mis)matches for each pregnancy was determined by randomization of the paternal HLA genotype. For this, it was first determined if a fetal allele was of maternal or paternal inheritance by deducing the fetal and maternal HLA genotype of the HLA-A, -B, -C, -DR, and -DQ loci. If the mother and child had identical heterozygous HLA genotypes, the classification between the maternally or paternally inherited gene was randomized. Consecutively, the paternally inherited genes of one combination were randomly divided in triplicate over the rest of the group. The expected values of the number of HLA (mis)matches of the three complete artificial fetal HLA genotypes, in relation to the maternal genotype, were calculated by direct counting. Eventually, the expected number of (mis)matches was determined as the average of the (mis)matches of the three artificial genotypes. The data of observed and expected HLA compatibility was analyzed per locus separately, as well as for HLA class I loci or for HLA class II loci combined, and for the total genotype of HLA alleles. The analysis described was repeated for the KIR/HLA-C combinations; maternally and paternally inherited HLA-C genotypes were randomly divided in triplicate over the cases and combined with the fixed KIR genotypes. Expected values were calculated for all possible KIR/HLA-C combinations and for paternally inherited HLA-C genotypes only.

The Chi-squared test was used to examine the differences between the expected and observed degree of HLA (in)compatibility and KIR/HLA-C combinations. The Bonferroni method was applied to correct p-values for multiple testing. Statistical analyses were performed using SPSS Statistics 25 (IBM SPSS Software) and GraphPad Prism 8. A p-value of <0.05 was considered statistically significant.

## Summary of Results

### Patient Characteristics

We studied 451 women with an uncomplicated pregnancy and 77 women who developed preeclampsia. The baseline characteristics of all included pregnancies are shown in **Table 1**. The gestational age, mode of delivery, highest diastole, birth weight and birth weight percentile were different in the group of pregnancies complicated with preeclampsia compared to the uncomplicated pregnancies, inherent to the clinical course of preeclampsia. The average maternal age and the median gravidity, parity and number of spontaneous miscarriages were higher in the uncomplicated pregnancies compared to pregnancies complicated by preeclampsia.

**Table 1:** Baseline Characteristics.

	Uncomplicated (n=451)	Preeclampsia (n=77)	P-value^a^
**Pregnancy**			
Gestational Age (d), mean ± SD (range)	277 *± 7.6 (266-297)*	224 *± 22.8 (170-283)*	<0.001*
Mode of Delivery, amount (%)			<0.001^#^
* Spontaneous*	172 *(38.1)*	18 *(23.4)*	
* Primary caesarean section*	221 *(49.0)*	44 *(57.1)*	
* Secondary caesarean section*	24 *(5.3)*	15 *(19.5)*	
* Unknown*	34 *(7.5)*	0 *(0)*	
Highest Diastole (mmHg), mean ± SD	78 *± 9.2*	105 *± 12.5*	0.003*
**Mother**			
Maternal Age (y), mean ± SD (range)	33 *± 4.6 (16-43)*	31 *± 5.5 (20-46)*	0.036*
Gravidity, median (range)	3 *(1-10)*	1 *(1-9)*	<0.001^$^
Parity, median (range)	1 *(0-6)*	0 *(0-5)*	0.001^$^
Spontaneous miscarriages, median (range)	0 *(0-9)*	0 *(0-3)*	<0.001^$^
**Child**			
Gender (%)			0.214^#^
* Female*	50.8	54.5	
* Male*	45.5	45.5	
* Unknown*	3.8	0	
Birth weight (gr), mean ± SD (range)	3541 *± 483 (1940-5285)*	1532 *± 813 (380-4030)*	<0.001*
Birth weight percentile, mean range	51-80	11-20	<0.001^#^
* Percentage under p<5, %*	2.0	14.3	

SD, standard deviation. ^a^Compared to uncomplicated pregnancies. *P-value calculated with independent samples t-test. ^#^P-value calculated with Chi-Square test. ^$^P-value calculated with Mann-Whitney-U test. p < 0.05 is considered significant.

### Genetic Frequencies

Of the uncomplicated pregnancies, the genotypic frequencies of the HLA alleles of mothers and children were tested for Hardy-Weinberg equilibrium (**Supplementary Table 1**). The maternal analysis of the HLA-A and -B locus showed significant deviation from the equilibrium with more A*02/A*11 and B*35/B*40 genotypes and less B*15 and B*35 genotypic combinations than expected-by-chance. For the fetal analysis of the uncomplicated pregnancies, all HLA loci were in Hardy-Weinberg equilibrium.

The samples size of the pregnancies complicated with preeclampsia was too small for the analysis of Hardy-Weinberg equilibrium. Thus, a conclusion of the random appearance of the genotypic frequencies of the HLA alleles cannot be drawn. The homozygous and heterozygous frequencies were, however, in balance as there were no significant differences.

### Maternal-Fetal HLA (Mis)Match Analysis

The amount of observed HLA (mis)matches was compared to the amount of expected HLA (mis)matches in both uncomplicated pregnancies and pregnancies complicated with preeclampsia.

In uncomplicated pregnancies, the distribution of the amounts of total HLA matches between mother and child was comparable to the amount expected-by-chance (**Table 2**). Likewise, there was no difference between the observed and expected-by-chance amount of maternal-fetal matches for individual HLA loci, HLA class I and HLA class II.

**Table 2:** Observed and expected-by-chance amount of HLA matches in uncomplicated pregnancies and pregnancies complicated with preeclampsia.

	Uncomplicated (n=451)							Preeclampsia (n=77)							P-value* Uncomplicated vs Preeclampsia (observed only)
	Observed(number of matches)			Expected(number of matches)			P-value*	Observed(number of matches)			Expected(number of matches)			P-value*	
	**1**	**2**		**1**	**2**			**1**	**2**		**1**	**2**			
***HLA-A***	86.0%	14.0%		88.2%	11.8%		0.244	85.7%	14.3%		88.3%	11.7%		1.000	1.000
***HLA-B***	91.6%	8.4%		92.9%	7.1%		0.271	87.0%	13.0%		93.5%	6.5%		0.098	0.097
***HLA-C***	85.8%	14.2%		86.9%	13.1%		0.485	77.9%	22.1%		89.6%	10.4%		0.007	0.025
***HLA-DRB1***	88.2%	11.8%		90.7%	9.3%		0.075	87.0%	13.0%		93.5%	6.5%		0.326	0.710
***HLA-DQB1***	81.4%	18.6%		83.1%	16.9%		0.314	77.9%	22.1%		84.4%	15.6%		1.000	0.382
* * ***Class I***	**3**	**>3**		**3**	**>3**			**3**	**>3**		**3**	**>3**			
	74%	25.9%		73.9%	26.1%		0.915	64.9%	35.1%		75.3%	24.7%		0.240	0.038
* * ***Class II***	**2**	**>2**		**2**	**>2**			**2**	**>2**		**2**	**>2**			
	78.7%	21.3%		79.4%	20.6%		0.727	75.3%	24.7%		81.8%	18.2%		1.000	0.366
* * ***Total***	**5**	**6**	**>6**	**5**	**6**	**>6**		**5**	**6**	**>6**	**5**	**6**	**>6**		
	59.2%	25.1%	15.7%	59.2%	27.7%	13.1%	0.166	48.0%	27.3%	24.7%	63.6%	24.7%	11.7%	0.012	0.021

*P-values calculated by Chi-square analysis. P-values are corrected for multiple comparisons with the Bonferroni method. p < 0.05 is considered significant.

In pregnancies complicated with preeclampsia, the total amount of HLA matches was differently distributed in observed pregnancies compared to values expected-by-chance (p= 0.012, **Table 2**), with an observed increase in the amount of >6 HLA matches. More specifically, the degree of maternal-fetal matching of HLA-C was higher in observed pregnancies than was expected-by-chance (p=0.007, **Table 2**).

When comparing observed number of HLA matches between uncomplicated and preeclamptic pregnancies, maternal-fetal compatibility of HLA-C (p=0.025), HLA class I (p=0.038) and total HLA matches (p=0.021) was differently distributed (**Table 2**, last column), with an overall tendency towards higher compatibility for the preeclamptic pregnancies.

A slight but significant difference was found in the distribution of the degree of maternal-fetal HLA class II mismatches in uncomplicated pregnancies (p=0.048, **Supplementary Table 2**), with an increase in both zero and two HLA class II mismatches. No differences were found between observed and expected-by-chance values of preeclamptic pregnancies in the analysis of the degree of HLA mismatches (**Supplementary Table 2**).

### Genotype and Allele Frequencies in Maternal-Fetal KIR/HLA-C Combinations

We investigated in both pregnancy cohorts whether certain combinations of maternal KIR and fetal HLA-C genotype were observed more frequently than what would be expected-by-chance. Overall analysis of KIR/HLA-C genotype combinations, including paternally inherited HLA-C alleles only, showed no differences in observed and expected-by-chance frequencies for both uncomplicated pregnancies and pregnancies complicated with preeclampsia (**Table 3**).

**Table 3:** Observed and expected-by-chance KIR/HLA-C combinations.

	*Uncomplicated (n=309)*		P-value* Observed vs Expected	*Preeclampsia (n=49)*		P-value* Observed vs Expected	P-value* Uncomplicated vs Preeclampsia (observed only)
	*Observed (%)*	*Expected (%)*		*Observed (%)*	*Expected (%)*		
***Genotype***							0.292
*KIR AA/HLA C1*	20.1	21.7	0.465	20.4	17.7	0.956	
*KIR AA/HLA C2*	15.9	14.2		22.4	21.1		
*KIR Bx/HLA C1*	65.4	65.4		59.2	61.9		
*KIR Bx/HLA C2*	45.3	45.0		53.1	54.4		
***Paternally inherited C1/C2 only***							0.045
*KIR AA/HLA C1*	14.6	14.5	1.000	10.2	8.8	0.977	
*KIR AA/HLA C2*	9.7	9.8		16.3	17.7		
*KIR Bx/HLA C1*	46.6	46.7		38.8	40.1		
*KIR Bx/HLA C2*	29.1	29.0		34.7	33.3		

*P-values calculated by Chi-square analysis. P-values are corrected for multiple comparisons with the Bonferroni method. p < 0.05 is considered significant.

Comparing the two cohorts, the distribution of the KIR/HLA-C genotype combinations with paternally inherited HLA-C only was different in preeclamptic pregnancies compared to uncomplicated pregnancies (p=0.045, **Table 3**). There is an observed tendency towards increased HLA-C2 presence in preeclampsia (**Table 3**), which is reflected in the allele and genotype frequencies of HLA-C as well (**Supplementary Table 3**). There were no significant differences in frequencies of HLA-C alleles or genotypes between uncomplicated and preeclamptic cases. The maternal KIR genotype frequencies in uncomplicated pregnancies were similar compared to preeclamptic pregnancies.

## Discussion

To investigate whether there is a preferential selection for HLA compatibility and specific KIR/HLA-C combinations in pregnancy, the present study analyzed observed and expected frequencies in cohorts of uncomplicated and preeclamptic pregnancies. As no significant differences were found in the amount of total maternal-fetal and locus-specific HLA matches compared to expected-by-chance, we conclude there is no selection for increased maternal-fetal HLA compatibility in uncomplicated naturally conceived pregnancies. We found a similar observation for KIR/HLA-C combinations in uncomplicated pregnancies. In comparison to uncomplicated pregnancies, pregnancies complicated with preeclampsia showed a different distribution of maternal-fetal HLA-C, HLA class I and total HLA matching, with an overall tendency towards higher compatibility. Furthermore, the distribution of the genotype-combinations with maternal KIR and paternally inherited HLA-C was different in preeclamptic pregnancies, with an overall tendency towards more HLA-C2, compared to uncomplicated pregnancies.

These findings suggest that a successful uncomplicated pregnancy does not require a deviation from the Hardy-Weinberg equilibrium for maternal-fetal HLA compatibility. Although we observed a slight significant difference in distribution of the observed amount of total HLA mismatching compared to expected-by-chance values, the percentages per degree of HLA mismatch did not show a trend towards more or less maternal-fetal compatibility. Research on the degree of optimal maternal-paternal HLA compatibility is limited and even more scarce for the maternal-fetal associations in uncomplicated naturally conceived pregnancies (18). Likewise, associations of maternal-fetal HLA compatibility with pregnancy complications such as recurrent pregnancy losses and preeclampsia were explored without definite conclusions (11, 12). Our results suggest there is no (need for) selection of an increased extent of total HLA matching between mother and child in uncomplicated naturally conceived pregnancies as it there is no difference than what would be expected according to the Hardy-Weinberg principle. To confirm this hypothesis, one could repeat the present study in a population with more closely genetically related individuals. In general, these populations might contribute to a better understanding in the way HLA compatibility and selection is involved in human reproduction.  (19)

In preeclamptic pregnancies, we found an increase in maternal-fetal total HLA, HLA class I, and HLA-C compatibility. A systematic review from Saftlas et al. on HLA sharing in pregnancy as a determinant of preeclampsia showed a lack of studies investigating maternal-fetal compatibility instead of maternal-paternal sharing (12). The three studies that included maternal-fetal analysis only considered HLA-A, -B, and -DR (12). A similar methodology was seen in subsequent studies (20). Overall, there was a reoccurring result of an association of maternal HLA-DR allogenicity with preeclampsia. This was confirmed by Creaenmehr et al., showing that maternal-fetal reciprocal allogenicity of HLA-DR is associated with the best pregnancy outcomes (21). These results point towards the occurrence of an optimal degree of compatibility in a successful pregnancy, which is consistent with our results. The largest cohort study to date on specific HLA alleles and the risk on preeclampsia did not include the analysis of HLA-C and fully split up the analysis of the amount of total maternal-fetal HLA matches (22). A study by Triche et al. did examine total HLA matching between mother and fetus and found an association between preeclampsia and a higher degree of compatibility for total HLA, HLA class I and HLA-A (23). The findings of total HLA and HLA class I matching are consistent with the current study, while we also show more specifically a higher amount of HLA-C matching in preeclamptic cases compared to expected-by-chance values and to uncomplicated pregnancies. In comparison to the study methodology followed by Triche et al., we included the analysis of intra-cohort randomization (as displayed in **Supplementary Table 1**), eliminating influence of cohort characteristics and thus inter-cohort differences.

The increase in maternal-fetal total HLA and HLA class I compatibility in preeclamptic cases seems to be mainly due to the difference in observed and expected-by-chance values of HLA-C matching. The percentage of two HLA-C matches was more than doubled compared to expected-by-chance, suggesting HLA-C compatibility between mother and child is associated with immunological processes that lead to preeclampsia. We consider two main types of maternal allorecognition to be involved, namely T cell responses to paternally-derived antigens and uNK cell interaction with fetal HLA-C through KIR receptors (1).

Firstly, HLA-C matched pregnancies have previously been linked to less maternal lymphocyte proliferation and absence of functional CD4^+^CD25^bright^ regulatory T cells at the maternal-fetal interface (10). The study, by Tilburgs and colleagues, suggested an indirect allorecognition of fetal HLA-C antigens by maternal CD4^+^ T cells (10). Disproportional populations of regulatory and effector T cells are a well-recognized features of preeclampsia (24).

Interestingly, the observed pattern of increased compatibility in preeclamptic pregnancies seems to be coherent with the HLA expression profile of the trophoblast. HLA-C is the only classical HLA class I molecule expressed by the EVT, while it completely lacks expression of HLA class II molecules (4). Interaction of HLA-C with KIR receptors on uNK cells inhibits their cytotoxic activity and modulates cytokine production and growth factors by uNK cells in favor of EVT invasion and placental vascular remodeling (8). Although the exact etiology is still unknown, principal characteristics of preeclampsia include disturbed spiral artery remodeling and poor placental development (25). Therefore, we expect our finding of an increased degree of maternal-fetal HLA-C compatibility in preeclamptic cases to be related to more self-HLA-C recognition by uNK cells and thus decreased activation of these cells.

The importance of uNK cell and trophoblast interactions is further underlined by the association of paternal HLA-C2 with the risk of preeclampsia (7). These findings were suggested to be based on the binding of HLA-C epitopes with KIR2DL1 on the A haplotype and KIR2DS1 on the B haplotype (7). Remarkably, the first suppresses and the second induces uNK cell responses involving placentation (26). The KIR A haplotype has a greater number of inhibitory KIR and lacks activating KIR, which might explain the association of preeclampsia and fetal growth restriction with the combination of KIR AA and C2 (8, 27). In our cohort, we observed a different distribution of the KIR/HLA-C genotype combinations with paternally inherited HLA-C in preeclamptic pregnancies compared to uncomplicated pregnancies. Similar to previous studies, our results show a tendency of increased presence of paternally inherited HLA-C2 (in combination with both KIR AA and KIR B) in preeclamptic cases when compared to uncomplicated pregnancies. A similar tendency for preeclamptic pregnancies was observed in HLA-C2 allele frequency in comparison to uncomplicated pregnancies.

In comparison to expected-by-chance values, no differences in observed combinations of maternal KIR and fetal HLA-C genotypes were found in either uncomplicated or preeclamptic pregnancies. These findings indicate that there is no preferential selection for particular KIR/HLA-C haplotype combinations in uncomplicated pregnancies. In oocyte donation pregnancies, similar conclusions were drawn as no favorable combination was found with respect to maternal KIR and fetal HLA-C (13). We expect that the association of preeclampsia with certain KIR/HLA-C combinations is based on specific (paternally inherited) genes instead of general haplotypes.

The biological mechanisms behind an optimal degree of maternal-fetal HLA (in)compatibility with associated successful pregnancy outcomes remains speculative. One possibility may be that a too high degree of compatibility may root a lack of involvement of maternal immune cells in the uterus, as discussed for both T cells and uNK cells. Interaction of uterine leukocytes with fetal trophoblasts is required for proper placentation and thus fetal growth. Furthermore, it is speculated that a prior and prolonged exposure to paternal antigens in seminal fluid stimulates a maternal immunological protective response to the semi-allogeneic fetus (23, 28). Induction of such immune response may be reduced if there is a high degree of HLA sharing between mother and father. Moreover, a significantly higher extent of total HLA compatibility in preeclampsia suggests a broader contribution than HLA-C or HLA class I alone. In uncomplicated pregnancies, HLA class II expression is epigenetically repressed (29). A recent study suggested expression of HLA-DR by syncytiotrophoblasts in placentae from pregnancies complicated with preeclampsia, potentially forming an immunogenic trigger towards the maternal immune system (30). Another (indirect) effect of immune recognition may be caused by microchimerism, leading to an interaction between paternally inherited fetal antigens and maternal leukocytes that involves incompatible HLA class II molecules as well. Interestingly, a higher degree of trafficking of fetal cells into the maternal periphery was observed in a study of preeclamptic cases, possibly contributing to a higher level of microchimerism (31). Thereby, the pathophysiological characteristic of the imbalance in effector and regulatory T cells in preeclampsia reinforces these findings (32).

Relevance of testing for Hardy-Weinberg equilibrium has been shown in multiple genetic association studies as well as in mendelian randomization research, as aberration from the equilibrium potentially points at inbreeding, population stratification or issues in typing (33–35). In our study, the Hardy-Weinberg analysis of the maternal HLA-typing resulted in a significant deviation from the equilibrium with more A*02/A*11 and B*35/B*40 genotypes and less B*15 and B*35 genotypic combinations than expected. As the population is compared to itself by randomization, the deviation from Hardy-Weinberg equilibrium can be explained by the distribution of ethnicity within the population or related families from the area. The abundance of the A*02/A*11 allele is as expected, as our population is predominantly Caucasian, the ethnic group with the most homogenous prevalence of the allele (36). The frequency of the B*35/B*40 genotype cannot be attributed to ethnicity as it is hardly investigated among different populations (37). The lack of B*15 and B*35 alleles can be explained by the fact that these haplotypes mostly occur in East-Asian populations (37).

In conclusion, this study is the first to show there is no need for the selection of increased maternal-fetal HLA compatibility in uncomplicated pregnancies. In contrast, preeclamptic pregnancies show a tendency of higher maternal-fetal HLA-C, HLA class I, and total HLA matching, as significantly different distributions of matching were observed compared to uncomplicated pregnancies. We further confirmed previous literature on the importance of maternal KIR and paternally inherited fetal HLA-C combinations in the occurrence of preeclampsia. These findings help to understand the immunological basis for a successful pregnancy and the pathophysiological mechanism of the highly occurring complication of preeclampsia, to eventually contribute to maternal and fetal risk assessment and therapy.

## Data Availability Statement

The raw data supporting the conclusions of this article will be made available by the authors, without undue reservation.

## Ethics Statement

The studies involving human participants were reviewed and approved by the ethics committee of the LUMC with protocol number P16.048. The patients/participants provided their written informed consent to participate in this study.

## Author Contributions

LH and NS analyzed data, performed statistical analysis and wrote the manuscript. LH extended and finalized the statistical analysis and writing of the manuscript. GH performed and supervised statistical analysis. CK performed sample work-up and organized the database. DR, LL and FC advised on and supervised writing the manuscript. MH and ME designed the study and are the primary editors and supervisors of the manuscript. All authors contributed to the article and approved the submitted version.

## Conflict of Interest

The authors declare that the research was conducted in the absence of any commercial or financial relationships that could be construed as a potential conflict of interest.
