# Supplementary material for: Maternal-Fetal HLA Compatibility in Uncomplicated and Preeclamptic Naturally Conceived Pregnancies
Source: Front Immunol. 2021 May 13;12:673131. doi: 10.3389/fimmu.2021.673131 (PMC8155594; doi:10.3389/fimmu.2021.673131)
Supplement: Supplementary file 1 [file DataSheet_1.docx]

**SUPPLEMENTARY DATA** as part of the

Manuscript for submission at Frontiers in Immunology, subject: Immune Regulations in Reproductive Organs and Organ Transplant

**Title**

Maternal-fetal HLA compatibility in uncomplicated and preeclamptic naturally conceived pregnancies

**Authors**

L.J. van ’t Hof^1^, N. Schotvanger^2^, G.W. Haasnoot^2^, C. van der Keur^2^, D.L. Roelen^2^, E.E.L.O. Lashley^1^, F.H.J. Claas^2^, M. Eikmans^2,†^, M.L.P. van der Hoorn^1,†^.

Affiliations

1. Department of Obstetrics and Gynaecology, Leiden University Medical Center, Leiden, the Netherlands

2. Department of Immunology, Leiden University Medical Center, Leiden, the Netherlands

†. These authors share last authorship

**Appendix A. Supplementary Data**

Supplementary Table 1. Analysis of Hardy-Weinberg equilibrium

|  | ***Locus*** | ***Uncomplicated (n=451), p-value*** | ***Preeclampsia (n=77)^#^,***  ***p-value*** | |
| --- | --- | --- | --- | --- |
|  |  |  | Homozygous^#^ | Heterozygous^#^ |
| ***Maternal*** | HLA-A | 0.0423* | *0.3563* | *0.7073* |
|  | HLA-B | 0.0356* | *0.2855* | *0.7156* |
|  | HLA-C | 0.2866 | *0.2554* | *0.5684* |
|  | HLA-DR | 0.0888 | *0.4300* | *0.7785* |
|  | HLA-DQ | 0.8955 | *0.7783* | *0.8772* |
| ***Fetal*** | HLA-A | 0.6690 | *0.6674* | *0.8574* |
|  | HLA-B | 0.7049 | *0.3531* | *0.7594* |
|  | HLA-C | 0.3887 | *0.6139* | *0.8217* |
|  | HLA-DR | 0.4724 | *0.9104* | *0.9680* |
|  | HLA-DQ | 0.3383 | *0.6806* | *0.8218* |

* significant deviation from equilibrium (p < 0.05)

^#^ The comparison with the Hardy-Weinberg equilibrium is impossible due to sample size. The homozygous and heterozygous frequencies are, however, in balance as there are no significant differences.

Supplemental Table 2. Observed and expected-by-chance amount of HLA mismatches in uncomplicated pregnancies and pregnancies complicated with preeclampsia.

|  | ***Uncomplicated***  *(n=451)* | | | | | | | | | ***Preeclampsia***  *(n=77)* | | | | | | | | | ***P-value****  ***Uncomplicated vs Preeclampsia***  ***(Observed only)*** |
| --- | --- | --- | --- | --- | --- | --- | --- | --- | --- | --- | --- | --- | --- | --- | --- | --- | --- | --- | --- |
|  | ***Observed***  *(number of mismatches)* | | | | ***Expected***  *(number of mismatches)* | | | | ***P-value**** | ***Observed***  *(number of mismatches)* | | | | ***Expected***  *(number of mismatches)* | | | | ***P-value**** |  |
|  | **0** | **1** |  |  | **0** | **1** |  |  |  | **0** | **1** |  |  | **0** | **1** |  |  |  |  |
| ***HLA-A*** | 29.5% | 70.5% |  |  | 27.1% | 72.9% |  |  | 0.244 | 27.3% | 72.7% |  |  | 26.0% | 74.0% |  |  | 1 | 0.584 |
| ***HLA-B*** | 19.3% | 80.7% |  |  | 16.0% | 84.0% |  |  | 0.054 | 19.5% | 80.5% |  |  | 13.0% | 87.0% |  |  | 0.811 | 0.898 |
| ***HLA-C*** | 32.8% | 67.2% |  |  | 31.3% | 68.7% |  |  | 0.507 | 36.4% | 63.6% |  |  | 29.9% | 70.1% |  |  | 1 | 0.495 |
| ***HLA-DRB1*** | 23.9% | 76.1% |  |  | 22.4% | 77.6% |  |  | 0.429 | 24.7% | 75.3% |  |  | 22.1% | 77.9% |  |  | 1 | 0.796 |
| ***HLA-DQB1*** | 42.6% | 57.4% |  |  | 42.6% | 57.4% |  |  | 1 | 42.9% | 57.1% |  |  | 41.6% | 58.4% |  |  | 1 | 0.936 |
| ***Class I*** | **<2** | **2** | **3** |  | **<2** | **2** | **3** |  |  | **<2** | **2** | **3** |  | **<2** | **2** | **3** |  |  |  |
|  | 21.1% | 33.3% | 45.7% |  | 18% | 34.4% | 47.7% |  | 0.228 | 18.2% | 40.3% | 44.6% |  | 15.6% | 36.4% | 48.1% |  | 1 | 0.431 |
| ***Class II*** | **0** | **1** | **2** |  | **0** | **1** | **2** |  |  | **0** | **1** | **2** |  | **0** | **1** | **2** |  |  |  |
|  | 21.7% | 23.1% | 55.2% |  | 18.6% | 27.7% | 53.7% |  | 0.048 | 24.7% | 18.2% | 57.1% |  | 19.5% | 26.0% | 55.8% |  | 1 | 0.430 |
| ***Total*** | **<3** | **3** | **4** | **5** | **<3** | **3** | **4** | **5** |  | **<3** | **3** | **4** | **5** | **<3** | **3** | **4** | **5** |  |  |
|  | 18.8% | 24.2% | 31.5% | 25.5% | 15.1% | 26.0% | 34.1% | 24.8% | 0.121 | 22.1% | 19.5% | 36.4% | 22.1% | 14.3% | 27.3% | 31.2% | 28.6% | 0.581 | 0.494 |

* P-values calculated by Chi-square analysis. P-values are corrected for multiple comparisons with the Bonferroni method. p<0.05 is considered significant.

Supplemental Table 3. HLA-C1 and -C2 genotype and allele Frequencies

|  | ***Uncomplicated*** *(n=451)* | ***Preeclampsia*** *(n=77)* | ***p-value* Uncomplicated vs Preeclampsia*** |
| --- | --- | --- | --- |
| ***Allele*** *(%)* |  |  |  |
| *Maternal* |  |  | 0.390 |
| *C1* | 62.1 | 58.4 |  |
| *C2* | 37.9 | 41.6 |  |
| *Fetal* |  |  | 0.121 |
| *C1* | 64.4 | 57.8 |  |
| *C2* | 35.6 | 42.2 |  |
| ***Genotype*** *(%)* |  |  |  |
| *Maternal* |  |  | 0.608 |
| *C1/C1* | 38.4 | 33.8 |  |
| *C1/C2* | 48.2 | 49.4 |  |
| *C2/C2* | 13.3 | 16.9 |  |
| *Fetal* |  |  | 0.241 |
| *C1/C1* | 41.2 | 31.2 |  |
| *C1/C2* | 44.2 | 53.2 |  |
| *C2/C2* | 14.2 | 15.6 |  |

* P-values calculated by Chi-square analysis. p<0.05 is considered significant.
